# Value of artificial ascites to assist thermal ablation of liver cancer adjacent to the gastrointestinal tract in patients with previous abdominal surgery

**DOI:** 10.1186/s12885-020-07261-x

**Published:** 2020-08-14

**Authors:** Qiannan Huang, Jianguo Li, Qingjing Zeng, Lei Tan, Rongqin Zheng, Xuqi He, Kai Li

**Affiliations:** 1 Department of Medical ultrasonics, Guangdong Key Laboratory of Liver Disease Research, The Third Affiliated Hospital of Sun Yat-sen University, Guangzhou, Guangdong Province 510630 PR China; 2 Department of Infectious Diseases, The Third Affiliated Hospital of Sun Yat-sen University, Guangzhou, Guangdong Province 510630 PR China

**Keywords:** Artificial ascites, Thermal ablation, Liver cancer, Gastrointestinal tract, Previous abdominal surgery

## Abstract

**Background:**

To evaluate the feasibility and effectiveness of artificial ascites to assist thermal ablation of liver cancer adjacent to the gastrointestinal tract in patients with previous abdominal surgery.

**Methods:**

Thirty-nine patients with a total of 40 liver malignant tumors were enrolled between January 2016 and June 2019. All had histories of hepatectomy, splenectomy, cholecystectomy, and intestinal surgery. The distance between the tumor and the gastrointestinal tract was < 5 mm. Normal saline was used as artificial ascites to protect the gastrointestinal tract during thermal ablation. The success rate of the procedure, incidence of major complications, and the technical efficacy of ablation were recorded. Patients were followed for local tumor progression (LTP), and overall survival (OS).

**Results:**

The use of artificial ascites was successful in 38 of the 40 procedures (95%). Major complications occurred in two of the 39 patients (5.1%) following the procedure. One was an intestinal fistula that occurred in a failed case and was associated with an infection. The other was a liver abscess that occurred in a successful case. The technical efficacy of ablation was 100% (40/40 procedures). The median follow-up was 16 months. The 1-, 2-, and 3-year LTP rates were 2.9, 5.7 and 5.7%. The 1-, 2-, and 3-year OS rates were 97.1, 86.8 and 69.5%.

**Conclusion:**

In patients with previous abdominal surgery, artificial ascites is feasible and effective for assisting thermal ablation of liver cancer adjacent to the gastrointestinal tract.

## Background

Liver cancer is the most commonly diagnosed cancer and the fourth leading cause of cancer deaths worldwide, half of which are estimated to occur in China [1, 2]. Abdominal surgery, liver transplantation, and local thermal ablation are curative treatments, and thermal ablation has been used for many years as an effective, safe, and minimally invasive treatment for patients with early-stage hepatocellular carcinoma (HCC) [3, 4]. When performed with curative intent, the target tumor is covered by an ablation zone that extends at least a 5 mm beyond the expected tumor margin to reduce the possibility of microscopic residual tumor foci [5]. Thermal energy may spread to surrounding organs while treating tumors located peripherally, which increases the risk of complications. Gastrointestinal perforation, which can result from thermal damage associated with ablation, has a reported incidence 0.06–0.7% [6–10]. Artificial ascites injected percutaneously into the perihepatic peritoneal space provides a thermal barrier separating the ablation zone from the gastrointestinal tract [11]. Kondo et al. reported that radiofrequency ablation (RFA) with artificial ascites was safe and effective for treating hepatic tumors abutting the gastrointestinal tract. The success rate of procedures including artificial ascites ranges from 78 to 92.7% [10, 12–17]. The use of artificial ascites may be limited by abdominal adhesions that prevent fluid from separating hepatic from gastrointestinal tissue in patients with histories of previous abdominal procedures including hepatectomy, splenectomy, cholecystectomy, and intestinal surgery [10, 11, 17, 18]. Studies of thermal ablation for liver cancer in patients with previous abdominal surgery are lacking. In our clinical experience, artificial ascites is easy to use and effective, and associated with minimal complications. This retrospective evaluated the feasibility and efficacy of using artificial ascites to assist thermal ablation for liver cancer adjacent to gastrointestinal tract in patients with previous abdominal surgery.

## Methods

### Patients

The study was approved by the Ethical Review Board of our hospital and was conducted following the ethical guidelines of the Declaration of Helsinki. The enrolled patients had received thermal ablation with curative intent at our hospital between January 2016 and June 2019. Those who were 18–80 years of age, with pathologically confirmed or clinically diagnosed hepatic malignant tumors, ultrasound images showing ≤5 mm between the tumor and the gastrointestinal tract, previous abdominal procedures including hepatectomy, splenectomy, cholecystectomy, or intestinal surgery. Patients allergic to the ultrasound contrast agent (UCA), without contrast-enhanced computed tomography (CECT) or contrast-enhanced magnetic resonance (CEMR) evaluation 1–3 months after ablation were excluded.

### Instruments

A cooled-tip RFA system (Covidien, Mansfield, MA, USA) and an electrode with a 3 cm internally-cooled tip were used. A 2450 MHz microwave generator (Kangyou, Nanjing, China) and internally-cooled microwave antenna were used for microwave ablation (MWA). A Mylab Twice ultrasound system with a CA541 1–8 MHz abdominal probe (Esoate, Italy) was used for ultrasound examination. Contrast-enhanced ultrasound (CEUS) was performed with real-time contrast-enhanced imaging and a mechanical index of < 0.05. Artificial ascites was delivered with a central venous catheterization set (Arrow, USA) and an 18 G percutaneous transhepatic cholangiography (PTC) needle (Hakko, Japan) for the abdominal puncture.

### Administration of artificial ascites

The relationship between the index tumor and the gastrointestinal tract and the distance of separation were determined before the procedure by B-mode ultrasound and computed tomography or magnetic resonance. The access point for the administration of artificial ascites was near the index tumor, and the catheter as placed between the index tumor and the abutting gastrointestinal tract. A subxiphoid puncture was used for tumors located in left liver, with the catheter placed under the liver. For tumors in segment 7/8, the puncture was under the right liver, and the catheter was placed before the right liver. If the tumor was in segment 5/6, the puncture was under the right liver and the catheter was placed under the liver.

**Fig. 1:**
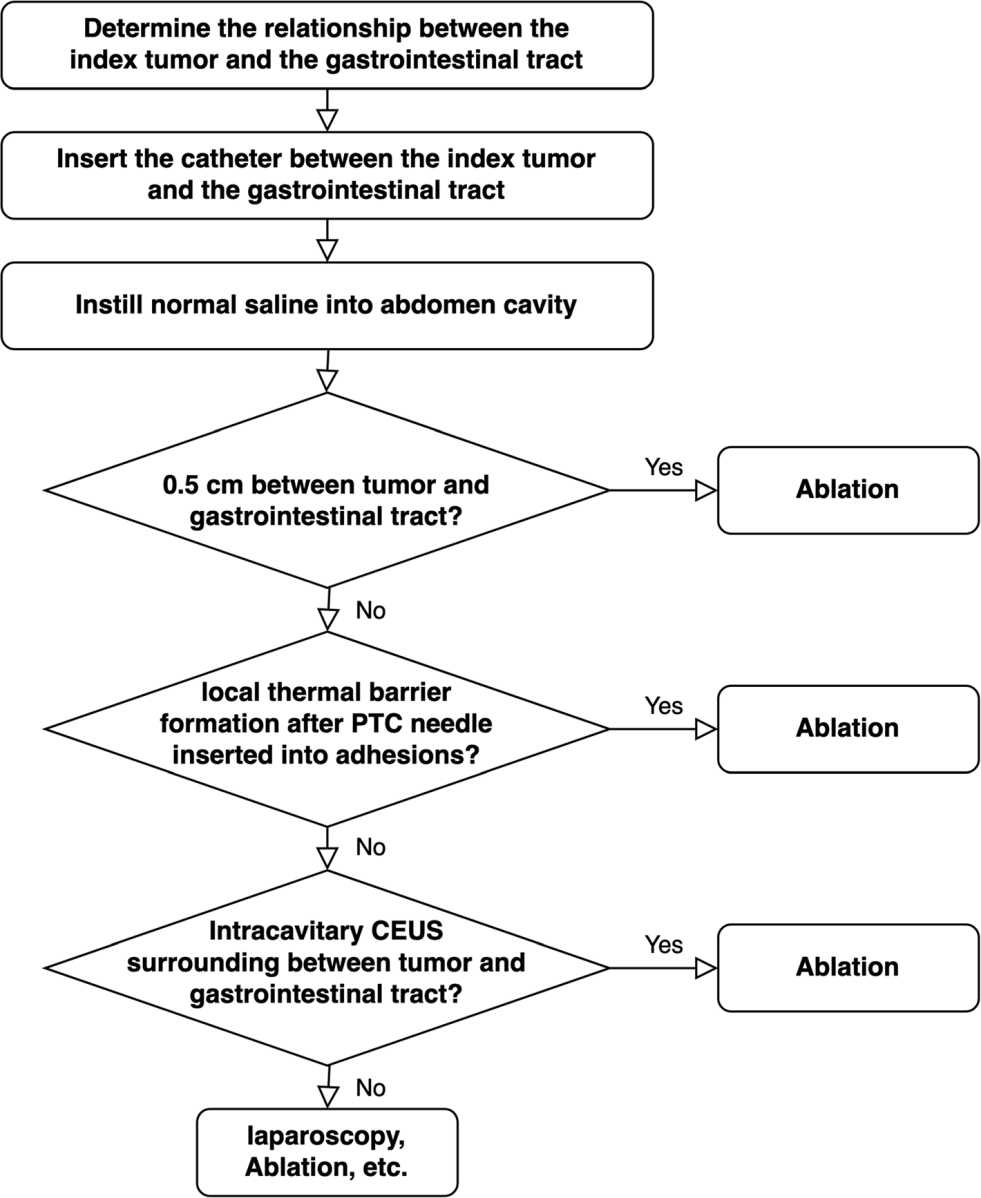
The schema of each step during procedure of administration of artificial ascites

**Fig. 2:**
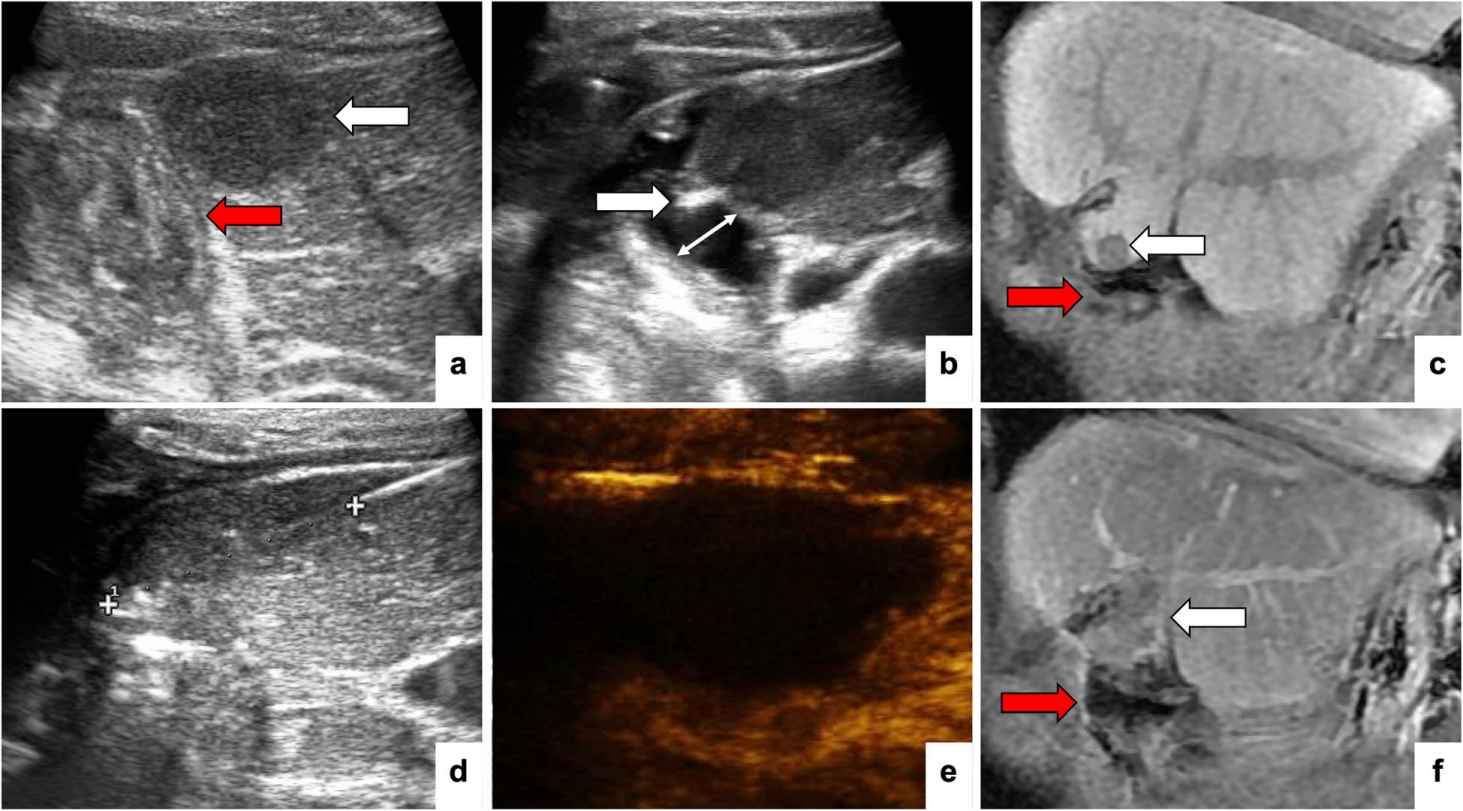
A patient with a history of hepatectomy and cholecystectomy. The index tumor was in segment 4 and ablated with RFA. Ultrasound (**a**) and MR (**c**) images show that the distance between the index tumor (white arrow) and intestine (red arrow) was < 5 mm. **b** A PTC needle is inserted with the tip (arrow) in the gap between the tumor and the intestine. Perfusion of normal saline established a local thermal barrier (double arrow line). **d** The RFA zone was about 3 cm × 2 cm and was measured 3 cm along the needle tract to control the size. After ablation, CEUS (**e**) and CEMR (**f**) show that the tumor was completely ablated

**Fig. 3:**
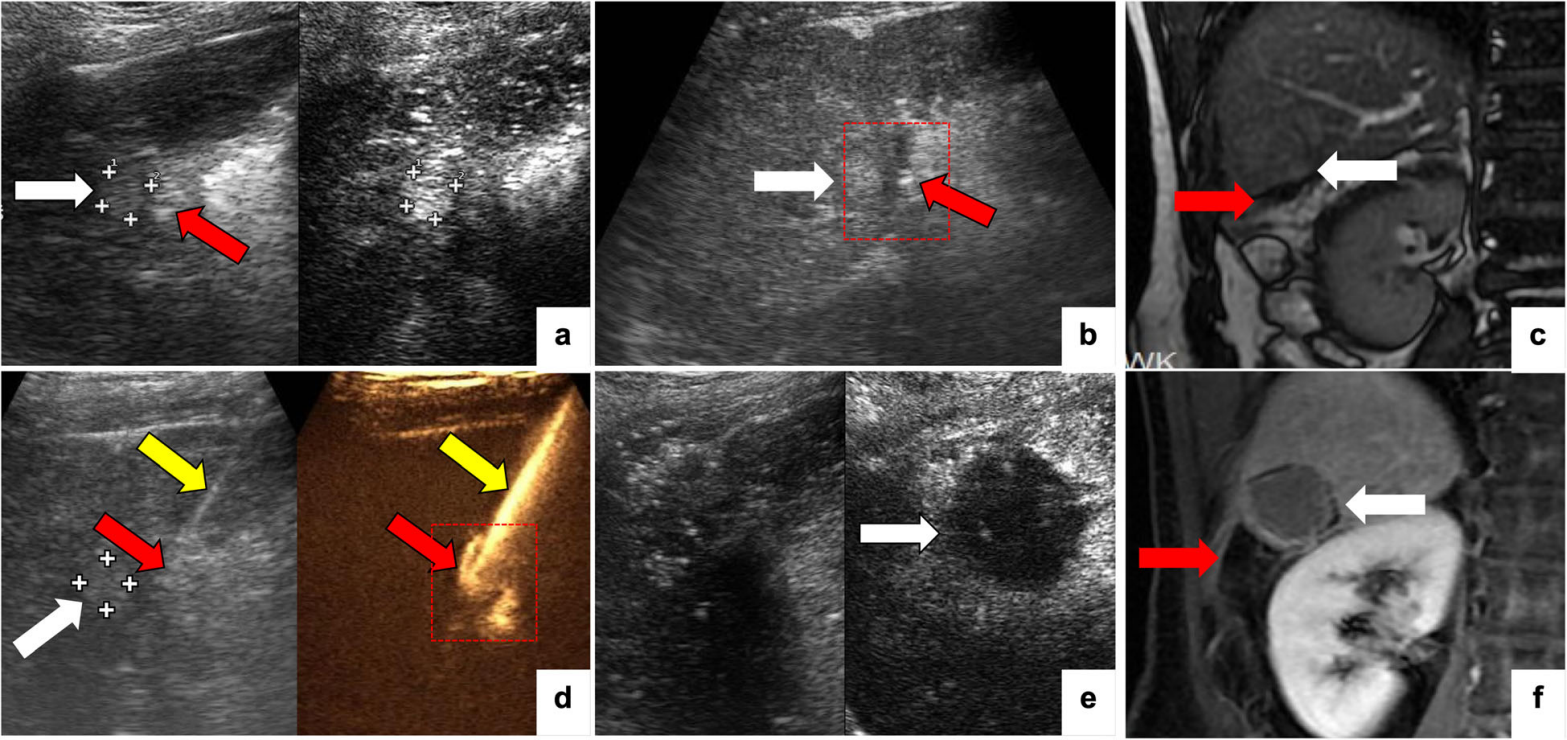
A patient with a history of hepatectomy and cholecystectomy. The index tumor was in segment 6 and was ablated with RFA. CEUS (**a**) and MR (**c**) show that the distance between the index tumor (white arrow) and intestine (red arrow) was < 5 mm. **b** A PTC needle was inserted with the tip visible (red arrow) in the gap between the tumor (white arrow) and the intestine. However, after perfusion with ascites, B-mode ultrasound showed that the gap could not be opened because of intraperitoneal adhesions (red outline). **d** Yellow arrow showed the tract of PTC needle. Intracavitary CEUS was injected through the PTC needle and showed ascites surrounding the index tumor and the intestinal tract. The ascites can flow continually between the lesion and the intestine (red outline), and continuing injection of normal saline removed the thermal energy induced by ablation. After ablation, CEUS (**e**) and CEMR (**f**) showed that the tumor was completely ablated

Ablation procedures were performed under general endotracheal anesthesia. The schema of each step during procedure of administration of artificial ascites was shown in Fig. 1. After induction, an 18 G PTC needle was inserted between the index tumor and the gastrointestinal tract under the guidance of ultrasound before inserting a guide wire and single-cavity central venous catheter. After catheterization, normal saline at room temperature was instilled into abdomen cavity to form a thermal barrier. Artificial ascites was rapidly infused by gently compression of the normal saline bag with a blood pressure cuff. If the index tumor was successfully separated by least 0.5 cm from the adjacent gastrointestinal tract, then ablation was performed. If adhesions were present between the tumor and the adjacent tissue, formation of a local thermal barrier was attempted using a PTC needle with gentle compression of the saline bag (Fig. 2). If a local thermal barrier could not be formed, then intracavitary CEUS was performed to observe the distribution of the artificial ascites. If ascites surrounded the index tumor and the gastrointestinal tract, then the injection of normal saline to cool the thermal energy induced by ablation was continued (Fig. 3). The success rates of each step of artificial ascites implementation were recorded. If intracavitary CEUS found that ascites did not surround the index tumor and separate it from the gastrointestinal tract, then laparoscopy was used to assist separation of the adhesions. However, when it is hard to separate the adhesions by laparoscopy after evaluating by surgeons, then ablation was performed with monitoring by B-mode ultrasound. The closest approach of the tip of electrode or antenna to the gastrointestinal tract was monitored to control the ablative zone and avoid gastrointestinal damage.

### Performance of thermal ablation

Ultrasound guided thermal ablation procedures were performed by three senior interventional physicians, each with more than 5 years of experience. The radiofrequency generator was set in impedance mode and maximum output. Each radiofrequency electrode insertion was for approximately 12 min. The ablative zone was about 3 cm × 2 cm. The microwave generator was set at 60 watts and each microwave antenna insertion was maintained for 6 min. The ablative zone was about 3.5 cm × 2.5 cm. The tumors were ablated following a previously determined plan with single or overlapping multiple insertions required to achieve a sufficient ablation zone. Complete ablation of the index tumor zone was evaluated by CEUS 5–10 min after completing the procedure, with supplementary ablation performed as necessary. If the non-perfusion zone completely covered the entire tumor, then the procedure was considered a technical success. CEUS was also used to observe the blood supply of the gastrointestinal tract and evaluate gastrointestinal damage.

### Postoperative observation and follow-up

Vital signs and clinical symptoms were monitored after ablation with ultrasound examination within 24–72 h to find any early complications. All major complications were recorded. The definition of major complication is an event that leads to sub-stantial morbidity and disability that increases the level of care, or results in hospital admission, or substantially lengthens the hospital stay [5]. Patients without severe complications were discharged 3–7 days after the procedure. Patients were evaluated by CECT or CEMR 1 month after the procedure, and if ablation was found technically effective at that time, then follow-up was repeated every 3 months. The technical efficacy, occurrence of major complications, local tumor progression (LTP), and over survival (OS) were recorded. Technique efficacy describes the achievement after the macroscopic tumor was completely ablated according to 1-month CECT or CEMR [5]. LTP refers to the appearance of tumor foci at the edge of the ablation zone, after at least one contrast-enhanced follow-up study has documented adequate ablation and an absence of viable tissue in the target tumor and surrounding ablation margin [5].

### Statistical analysis

Statistical analysis was performed with SPSS 25.0 (IBM Corp., Armonk, NY, USA). Continuous data were reported as means ± standard deviation if they were normally distributed or as the medians (range) if they were not normally distributed. Enumeration data were reported as numbers and percentages. The OS and LTP were estimated by the Kaplan-Meier method. Differences with *P*-values < 0.05 were considered significant.

## Results

### Enrollment

A total of 39 patients with 40 liver malignant tumors were enrolled between January 2016 and June 2019. The patient and tumor characteristics are shown in Table 1.

**Table 1:** Patient and tumor characteristics

Characteristics	Total number
Gender (Male/Female)	36/3
Age (mean ± SD)	53 ± 10.9 (25 ~ 74)
Liver cirrhosis (Yes/No)	30/9
Child-Pugh class (A/B)	38/1
No. of tumors (solitary/multifocal)	38/1
Treatment history:Hepatectomy/Cholecystectomy/Splenectomy/ Hepatectomy + Cholecystectomy/ Cholecystectomy + Splenectomy/ Hepatectomy+ Cholecystectomy + Splenectomy/ Transplantation/ Intestinal surgery	11/2/3/17/1/1/1/3
Diagnosis (HCC/ICC/ Metastasis)	32/4/4
Tumor diameter (median, range)	18,10–50
Tumor diameter (> 30 mm/<=30 mm)	7/33
Segment (I/II/III/IV/V/VI/VIII)	1/9/5/4/11/9/1
Index tumor located at the same side of the Hepatectomy^a^ (Yes/No)	16/13
Ablation method (RFA/WMA)	36/4

Index tumor located at the same side of the hepatectomy^a^ was defined as the liver resection site located in the left or right lobe of the liver, and the index tumor on the same side of the lobe as the resection site

*HCC* Hepatocellular carcinoma, *ICC* Intrahepatic cholangiocarcinoma, *RFA* Radiofrequency ablation, *MWA* Microwave ablation

### Success of artificial ascites implementation

Abdominal puncture and catheterization were performed in all 39 patients and artificial ascites was successfully delivered in procedures involving 38 of the 40 tumors (95%). The median volume of artificial ascites was 900 (60–3500) ml. Two tumors were not separated from the gastrointestinal tract by the artificial ascites, one of which was ablated with laparoscopic assistance and the other was ablated under strict monitoring by ultrasound and CEUS. The duration of the ablation procedures was 97 (28–355) min.

Twenty-nine patients had a history of hepatectomy. Fifteen of the 16 cases (93.8%) with tumor ablation on the same side as the hepatectomy and all of the 13 tumors located at a different site than the previous hepatectomy (100%) were successfully treated using artificial ascites. The difference was not significant (*P* = 1.000).

### Complications

There were no treatment-related deaths or cardiopulmonary complications caused by volume overload. Diuretics or paracentesis were not required to manage any patient with infused artificial ascites. Two major complications occurred in the 39 patients (5.1%). One patient with failed implementation of artificial ascites experienced an intestinal fistula and infection. The patient was 74 years old with diabetes. She had a previous history of gallbladder malignancies and underwent open cholecystectomy and hepaticojejunostomy. She had tried systemic chemotherapy but could not tolerate it. One year after surgery, she had liver metastases and received TACE. At this check, she was found two liver lesions and the bigger one was near the gastric cardia. The tumor had a maximum diameter of 4.5 cm, and proved to be difficult to separate the adhesions with laparoscopic assistance. After abdominal puncture and catheterization, intracavitary CEUS confirmed failure to separate the adhesion. Ablation was performed with ultrasound guidance. An intestinal fistula and local infection developed in the ablative area and resolved with percutaneous drainage and anti-infective therapy. The other complication was a liver abscess that occurred in a successful artificial ascites case. The patient had a history of splenectomy. The tumor was located in segment VI near the intestine and had a maximum diameter of 1.1 cm. The tumor was successfully separated from gastrointestinal tract with 300 ml artificial ascites. One month after ablation, the patient developed a liver abscess that resolved with percutaneous drainage and anti-infective therapy.

The most common minor complications after ablation pain and fever. Post-treatment fever of more than 38.5 °C was observed in 8 patients, and treated symptomatically with antipyretics. After treatment, 12 patients required the administration of analgesics after treatment. There were no local grounding pad burns or local hematomas.

### Follow-up and survival

**Fig. 4:**
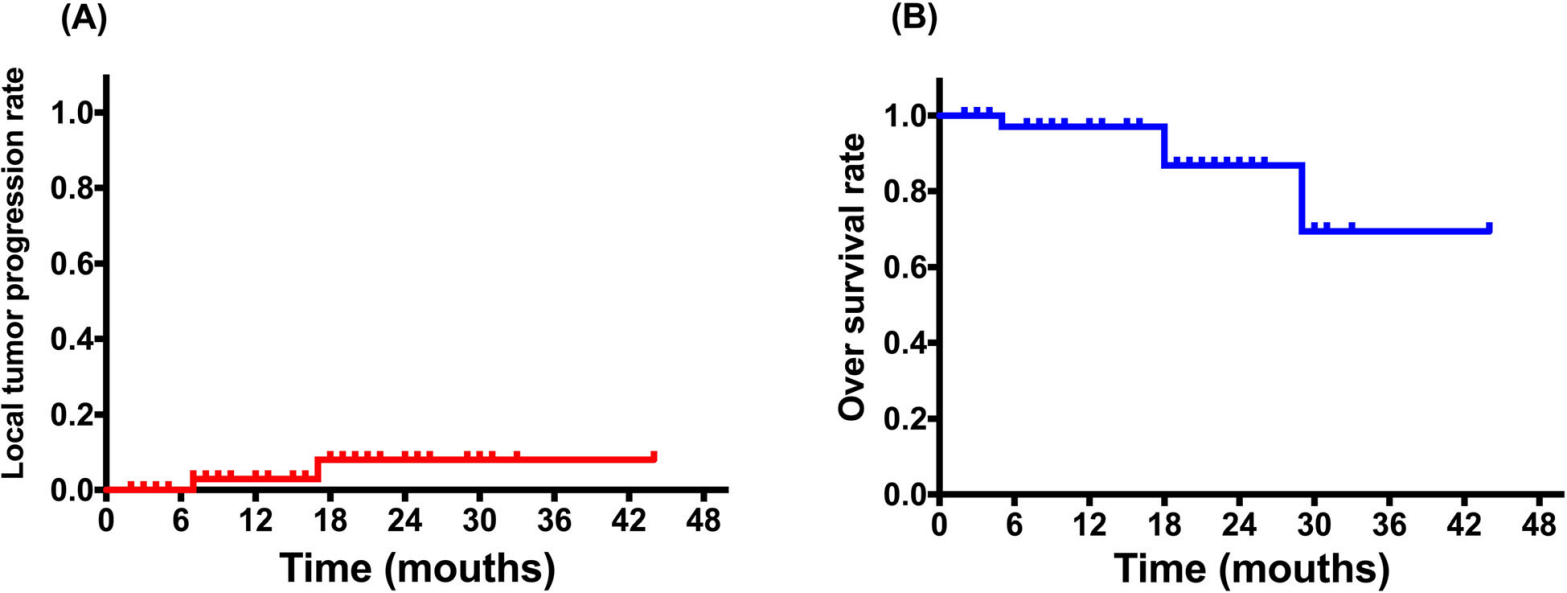
Survival curves. **a** The LTP rates were 2.9, 5.7 and 5.7% at the 1-, 2-, and 3-year time points, respectively. **b** The OS rates were 97.1, 86.8 and 69.5% at the 1-, 2-, and 3-year time points, respectively

Median follow-up time was 16 months. All patients were evaluated by CECT/CEMR within 1–3 months after the ablation procedure. All tumors were completely ablated; the technical efficacy rate of ablation was 100%. LTP occurred in two patients with successful implementation of artificial ascites. The 1-, 2-, and 3-year LTP rates were 2.9, 5.7 and 5.7% (Fig. 4a). Four patients died during follow-up, because of tumor progression. The 1-, 2-, and 3-year OS rates were 97.1, 86.8 and 69.5% (Fig. 4b).

## Discussion

Previous studies have reported that a history of abdominal surgery was the main reason to technical failure of artificial ascites because postoperative lesions were present in up to 93% of the patients [10, 11, 17, 18]. The difficulty of separating adhesions can increase the volume of the artificial ascites and the range of liver movement. Any movement of the omentum and gastrointestinal tract in relation to the index tumor can increase the difficulty of percutaneous catheterization. Percutaneous balloon catheters have been used to separate the index tumor from gastrointestinal tract, but they can be difficult to position, especially in patients with tumors distant from body surface [19, 20]. This method also requires special instruments that complicate its adoption. Laparoscopy can assist ablation, but it is more costly and invasive than artificial ascites and postoperative adhesions may be more likely [21]. A small dose of ethanol injected into the marginal tissue of the tumor can reduce thermal damage of the gastrointestinal tract but it is not a curative treatment [22, 23]. Artificial ascites is a relatively safe and effective option for tumors abutting the gastrointestinal tract. Further study is needed to confirm the usefulness and clinical benefit of this procedure to assist in local ablation of hepatic tumors in patients with previous abdominal surgery.

CT can clearly show the anatomical relationship between the lesion and the surrounding structure. It is believed by some clinicians that treatment under the CT control is safer. However, we used ‘ablation, Gastrointestinal, liver’ as keywords and searched on PubMed, and we did not find any literatures clearly stated that hepatic lesions must be ablated under CT control, or ultrasound-guided ablation will increase the incidence of complications. Besides, There are two literatures compared the application of CEUS/US and CT guided RFA for hepatocellular carcinoma, the results showed that there was no comparative difference in the incidence of complications between the two groups [24, 25]. Most articles using artificial ascites to assist ablation of liver cancers adjacent to gastrointestinal tract were conducted under the guidance of US, because it has the advantage of real-time monitoring, which is conducive to the formation of artificial ascites, and can real-time and accurately assess the depth and the position of needle [10, 12–17, 26]. Thus, we consider that ultrasound-guided artificial ascites assisted thermal ablation is an option for liver cancer tumors adjacent to gastrointestinal tract for in patients with previous abdominal surgery.

Artificial ascites was successfully implemented in 95%, of the cases in this study, which is consistent with previous reports of from 78 to 92.7% success [10, 12–17]. The success rate of artificial ascites implementation was improved by some modifications implemented before and during this study. The puncture site for instilling the artificial ascites was near the tumor, and the catheter was located between the index tumor and adjacent gastrointestinal tract if possible to ensure the accumulation of liquid in the space between them. An intravenous catheter was used because it is soft and can maintain the saline infusion and its position during ablation without real-time monitoring. If artificial ascites did not accumulate between the tumor and adjacent gastrointestinal tract an 18 G PTC needle was placed between them and liquid as perfused to form a local thermal barrier. Direct injection of UCA through the needle or catheter facilitates confirmation of correct needle or catheter position and the cavity morphology [27]. That allowed use of intracavitary CEUS to evaluate the distribution of artificial ascites. If liquid was present between the tumor and adjacent gastrointestinal tract, then perfusion could continue to remove thermal energy from the site. The study results confirm that these modifications in the use of artificial ascites were successful in separating the index tumor and gastrointestinal tract in more than 90% of the patients. Artificial ascites was a feasible technique to assist thermal ablation for liver cancer adjacent to the gastrointestinal tract in patients with previous abdominal surgery.

Mesothelial damage caused by inflammation or surgical trauma can trigger the formation of postoperative adhesions during peritoneal wound healing [21, 28]. Adhesions likely to form between an incision and the adjacent gastrointestinal tract. In this study there were 29 patients with a history of hepatectomy, but there were no significant differences in the successful implementation of artificial ascites in those with the index tumor on the same side or different side as the hepatectomy. The result shows that, in either situation, the adhesion could be broken and did not influence the distribution of the artificial ascites.

Major complications were associated with 5.1% of the study procedures, which is higher than reported in previous studies [10, 12–15, 17, 29]. One of the two complication cases experienced an intestinal fistula accompanied by infection that developed in a failed implementation of artificial ascites. The tumor had a maximum diameter > 3 cm. The large ablative zone might have increased the risk of gastrointestinal tract injury because the artificial ascites failed to separate the liver from the gastrointestinal tract. However, it was difficult to distinguish the intestinal fistula caused by infection or ablation. We considered that avoiding infection may be help reduce complications. Besides, when the artificial ascites cannot be performed, accurate calculation of the ablative zone may help complete the ablation, but the process may be restricted. In such cases, laparoscopy, laparotomy, or ethanol ablation might be helpful.

Some studies considered that saline can conduct electrical current due to its ionic composition, which may lead to non-target tissue heating while using RFA. However, based on our previous experience [30, 31], we did not have any complications associated with the choice of normal saline as artificial ascites. In addition, we did not find any articles about the complications of using normal saline as artificial ascites in human beings. We think it may be related to the following factors. Firstly, the RFA does not heat the normal saline directly, thus may less likely to conduct electrical current. Secondly, most cases of artificial ascites in our study was floating, which may reduce the local energy accumulation. Besides, some patients in our study have diabetes and were not suitable for D5W. Artificial ascites has been reported to increase the risk of intraperitoneal bleeding and tumor seeding because it washes coagulation substances away from the puncture site and decreases the pressure of the abdominal wall against the liver, facilitating the dissemination of tumor cells [10]. Bleeding and tumor seeding were not observed in this study. Cauterizing the needle track when withdrawing the electrode or antenna may help to prevent these complications [32]. As in previous reports, residual ascites disappeared spontaneously without additional diuretics or paracentesis.

All tumors in this study were ablated completely. The technical efficacy rate of ablation was 100%, indicating that artificial ascites was effective in to assisting thermal ablation. The 1-, 2-, and 3-year LTP rates were 2.9, 5.7 and 5.7%, and the 1-, 2-, and 3-year OS rates were 97.1, 86.8 and 69.5%. Both were in line with previous studies and a the experience at the study center, and support the use of artificial ascites can help achieve an effective therapeutic effect.

The study limitations include its single-arm retrospective design. A controlled trial should be designed and conducted, Secondly, the sample size was small. Thirdly, as the median follow-up was 16 months, additional monitoring is needed to better support the therapeutic benefit. Consequently, further research is needed to validate the clinical value of artificial ascites implementation in patients with a history of abdominal surgery.

## Conclusions

In conclusion, artificial ascites was feasible and effective to assist thermal ablation of liver tumors adjacent to gastrointestinal tract in patients with previous abdominal surgery. If artificial ascites fails to separate the liver from the gastrointestinal tract, auxiliary methods including laparoscopy, laparotomy, or ethanol ablation, can be considered as alternatives.

## Data Availability

The datasets used and analyzed during the current study are available from the corresponding author on reasonable request.

## Abbreviations

HCC: Hepatocellular carcinoma
RFA: Radiofrequency ablation
CECT: Contrast-enhanced computed tomography
CEMR: Contrast-enhanced magnetic resonance
MWA: Microwave ablation
CEUS: Contrast-enhanced ultrasound
UCA: Ultrasound contrast agent
PTC: Percutaneous transhepatic cholangiography
LTP: Local tumor progression
OS: Overall survival
